# Supplementary material for: Evaluating the frequency of English language requirements in clinical trial eligibility criteria: A systematic analysis using ClinicalTrials.gov
Source: PLoS Med. 2021 Sep 14;18(9):e1003758. doi: 10.1371/journal.pmed.1003758 (PMC8439488; doi:10.1371/journal.pmed.1003758)
Supplement: S1 Tables — Table A: ClinicalTrials.gov advanced search criteria. Table B: ClinicalTrials.gov funder type classification. (DOCX) [file pmed.1003758.s001.docx]

**Supplementary Tables**

Table A: ClinicalTrials.gov Advanced Search Criteria

Table B: ClinicalTrials.gov Funder Type Classification

**Table A: ClinicalTrials.gov Advanced Search Criteria***

| **Condition or Disease** | **Date Range** | **Number of Trials** |
| --- | --- | --- |
| Depression | 01 February 2017 to  15 September 2020 | 67 |
| Diabetes | 01 July 2017 to  15 September 2020 | 85 |
| Breast Cancer | 01 January 2016 to  15 September 2020 | 70 |
| Prostate Cancer | 01 January 2015 to  15 September 2020 | 55 |
| Infectious Disease (COVID-19) | 01 January 2019 to  01 December 2020 | 1023 (n = 611 COVID-19) |
| N/A | 01 January 2019 to  31 December 2019 | 7477 (n =288 with protocol) |
| N/A | 01 January 2020 to  01 December 2020 | 6890 (n = 78 with protocol) |

***All advanced search criteria listed are in addition to (1) Study Type: Interventional, (2) Age Group: Adult and Older Adult, and (3) Location: US.

**Table B: ClinicalTrials.gov Funder Type Classification**

| **ClinicalTrials.gov Registration Field** | **ClinicalTrials.gov Outcome Elements** | **Coded Outcome Elements** |
| --- | --- | --- |
| Funded By | U.S. Fed | Federal |
|  | NIH |  |
|  | U.S. Fed \| NIH \| Other |  |
|  | U.S. Fed \| NIH |  |
|  | U.S. Fed \| Other |  |
|  | NIH \| Other |  |
|  | Industry | Industry |
|  | Industry \| Other |  |
|  | Industry \| U.S. Fed | Federal/Industry  (Excluded from analysis) |
|  | Industry \| NIH |  |
|  | Industry \| NIH \| U.S. Fed |  |
|  | Industry \| U.S. Fed \| NIH \| Other |  |
|  | Industry \| NIH \| Other |  |
|  | Industry \| U.S. Fed \| Other |  |
|  | Other | Other  (Excluded from analysis) |
