# Supplementary material for: Evaluating the frequency of English language requirements in clinical trial eligibility criteria: A systematic analysis using ClinicalTrials.gov
Source: PLoS Med. 2021 Sep 14;18(9):e1003758. doi: 10.1371/journal.pmed.1003758 (PMC8439488; doi:10.1371/journal.pmed.1003758)
Supplement: Attachment — Submitted filename: 2021-06-15b PLOS Medicine response letter.docx [file pmed.1003758.s002.docx]

Response to *PLOS Medicine*

Manuscript ID PMEDICINE-D-21-01563R1

To the Editors and Reviewers,

Thank for your thoughtful consideration of our manuscript, and for the detailed and expert review. We agree with the majority of the comments and the recommendations made, and we appreciate the enthusiasm of the reviewers. While the constraints of length do limit our ability to fully address the reviewers’ comments, we have endeavored to revise the piece as suggested, and provide a point-by-point response below. We have annotated the changes and additions made to the manuscript and references, and these are indicated in track changes in the revised submission. Direct quotes from the editors and reviewers are numbered followed by the authors’ *Response*.

EDITORS

1. Please revise your title according to PLOS Medicine's style. Your title must be nondeclarative and not a question. It should begin with main concept if possible. Please place the study design (e.g."A cross-sectional study" or “systematic review”) in the subtitle (i.e., after a colon).

*Response:*

Changed as suggested. The revised title is:

Evaluating the frequency of English language requirements in clinical trial eligibility criteria: a systematic analysis using ClinicalTrials.gov

2. Abstract summary - At this stage, we ask that you reformat your non-technical Author Summary. The Author Summary should immediately follow the Abstract in your revised manuscript. This text is subject to editorial change and should be distinct from the scientific abstract. The summary should be accessible to a wide audience that includes both scientists and non-scientists.

*Response*:

As requested, we have added an Author Summary following the Abstract.

**Author Summary**

**Why was this study done?**

- Some clinical trials in the US exclude individuals who do not read, speak, or write English.
- While requiring English language proficiency for entry into clinical trials may sometimes be scientifically or ethically justified, often it is not, raising concerns of equity and justice.
- We sought to establish the frequency of English language requirements in clinical trials in the United States.

**What did the researchers do and find?**

- We reviewed 14,367 US clinical trials registered on ClinicalTrials.gov in 2019 and 2020.
- Of the 14,367 clinical trials, 18.98% (n=2,727) had English language requirements, and 2.71% (n=390) mentioned accommodation of a language other than English.
- Clinical trials funded by the federal government were more likely to require English than clinical trials funded by the life sciences and pharmaceutical industries.
- Compared to all clinical trials in 2019 and 2020, clinical trials related to COVID-19 and other infectious diseases were less likely to have English language requirements.
- In a separate analysis of clinical trials in four therapeutic areas (depression, diabetes, breast cancer, prostate cancer), trials related to depression were the most likely to require English (52.24%).

**What do these findings mean?**

- A meaningful percentage of clinical trials in the United States exclude individuals who do not speak, read, or write English.
- Clinical trials can be made more inclusive by eliminating language requirements that are not scientifically or ethically justified.

3. In the abstract Methods and Findings, please revise the subheading “Methods/Finding” to “Methods and Findings,”

*Response*:

Modified.

4. [In the abstract Methods and Findings,] [p]lease ensure that all numbers presented in the abstract are present and identical to numbers presented in the main manuscript text

*Response*:

We have reviewed for consistency, and we hope we have not overlooked any mistakes.

5. [In the abstract Methods and Findings,] [p]lease quantify the main results with both 95% CIs and p values

*Response*:

Changed as suggested throughout.

6. In the last sentence of the Abstract Methods and Findings section, please describe the main limitation(s) of the study's methodology

*Response*:

We have added the main limitations of the study’s methodology to the Abstract. We have also embedded these limitations in the manuscript text.

Abstract Excerpt:

One limitation of this study is that the computer program only searched for the terms ‘English’ and ‘Spanish’ and may have missed evidence of other language accommodations. Another limitation is that we did not differentiate between requirements to read English, speak English, understand English, and be a native English speaker; we grouped these requirements together in the category of English language requirements.

Manuscript Excerpt:

There are two limitations to the study methodology. One limitation is that the computer program only searched for the terms ‘English’ and ‘Spanish;’ if neither of these terms were identified, and a protocol was not available for manual review, language requirements that did not use the terms ‘English’ or ‘Spanish’ may have been overlooked. Another limitation is that the requirements to read, speak and/or understand English and/or be a native English speaker were grouped together in the category of English language requirements.

7. Did your study have a prospective protocol or analysis plan? Please state this (either way) early in the Methods section. If a prospective analysis plan (from your funding proposal, IRB or other ethics committee submission, study protocol, or other planning document written before analyzing the data) was used in designing the study, please include the relevant prospectively written document with your revised manuscript as a Supporting Information file to be published alongside your study, and cite it in the Methods section. A legend for this file should be included at the end of your manuscript. If no such document exists, please make sure that the Methods section transparently describes when analyses were planned, and when/why any data-driven changes to analyses took place. In either case, changes in the analysis-- including those made in response to peer review comments-- should be identified as such in the Methods section of the paper, with rationale.

*Response*:

The study did not have a prospective written protocol, but we did design a prospective plan for data analysis and amend the plan during the course of the research. We have revised the Methods section to describe the prospective data analysis plan and when and why we amended the analysis plan.

Excerpts:

Data analysis followed a prospective analysis plan

…(3) mentions that participants must be able to provide informed consent and/or communicate with study staff via phone call or email, or (4) does not mention any language requirement for participation (“no mention”). After reviewing some of the clinical trials in the sample, we determined that the ability to provide informed consent is independent of language and that there was no relevant distinction between categories (3) and (4) and therefore these two categories were pooled for further analysis.

In order to establish a relationship between the accommodation of a non-English language and increased diverse representation in clinical trials, we reviewed the ethnicity data in the trials with an actual start date between 01 January 2019 and 01 December 2020 that had posted results to ClinicalTrials.gov.

8. In the Methods and Results section, [p]lease provide 95% CIs and p values for estimates in the main text and tables

*Response*:

Changed as suggested throughout.

9. [In the Methods and Results section,] [w]hen a p value is given, please specify the statistical test used to determine it.

*Response:*

Changed as suggested.

Excerpt:

P-values were calculated using a two-tailed z-test of two proportions at significance level 0.001.

10. [In the Methods and Results section,] [p]lease do not report P<0.01; report as P < 0.001

*Response*:

Changed as suggested.

11. The presentation in table 3 is confusing. Please remove “n(%)” from the rows and instead write it at the top of the last 2 columns (“Requires English” and “Accommodates translation”).

*Response*:

Changed as suggested (Table 3). We have also edited table 2 (Table 2) to be consistent with this format.

12. Please define the following abbreviations in your tables 1: ICF, ASL

*Response*:

Changed as suggested (Table 1)

13. In table 1, please replace "subject" with participant, patient, individual, or person

*Response*:

Changed “subjects” to “participants.” (Table 1)

14. Please look separately at Spanish in the studies since that is the second most frequently spoken language and note how often that is mentioned/accommodated.

*Response*:

We appreciate the editors’ attention to the frequency of Spanish accommodation in clinical trials given the prevalence of individuals who communicate in Spanish in the United States. We have noted this analysis in the Methods and Findings section, tallied the number of clinical trials that accommodate Spanish, and added columns in table 2 (Table 2) and table 3 (Table 3) to show the number and percentage of clinical trials that accommodate Spanish.

Excerpts:

Since Spanish is the second most common language in the United States, we noted the number of trials that specifically mention the accommodation of Spanish as a subcategory of category (2).

15. Please use the "Vancouver" style for reference formatting and see our website for other reference guidelines

*Response*:

We have reviewed and revised the references to be consistent with Vancouver style. Please let us know if there are any errors in the reference formatting.

REVIEWER 1

1. While what was done isn't really wrong, I don't think it's ideal I am concerned that over three-quarters of the studies did not mention translation. This is a kind of missing data problem, but the usual methods of multiple imputation are likely to fail, since so much data was missing. Therefore, rather than use z-tests of two proportions, I would a) Simply look at the proportion that do accommodate translation and b) use multinomial logistic regression to examine whether the variable "translation" (with three levels) varies by any other trait you are interested in. This treats "missing" as its own category, which is, I think, a better approach than simply ignoring them.

*Response*:

Reviewer 1 suggests a different approach to data analysis, and we understand the rationale for suggesting using multinomial logistic regression. However, we do not agree with the characterization of trials that do not mention language requirements as ‘missing.’ There is no expectation that clinical trial eligibility criteria will contain language requirements or accommodations, so it is inaccurate to characterize the absence of language requirements or accommodations as ‘missing.' Only affirmative requirements are given in eligibility criteria—the absence of any criterion is not “missing.” As an example, a trial intended for women would include that criterion. One would not anticipate that every *other* trial would state “Must be male or female,” or “either male or female sex eligible.” If a trial has no eligibility criterion for renal function, one would not expect inclusion criteria to list “Any value for creatinine is acceptable.” In the same way, if no language is specified, it is not a relevant criterion and would not be mentioned; only the affirmative (either inclusion “must be able to read, write, and/or speak English” or exclusion “inability to read, write, and/or speak English”) criterion is informative. We are interested in comparing the proportions of clinical trials that require English (or specifically accommodate translation) and found that using z-tests of two proportions was most appropriate.

REVIEWER 2

1. This is a well-written and interesting paper that addresses an important issue in clinical trial eligibility. Strengths of the paper include a thoughtful introduction highlighting the importance of removing barriers to research participation to increase diversity in clinical trials and the use of the use of a rigorous and reproducible search strategy to identify language requirements for clinical trials with at least one US site. The main findings include 19.0% of trials on ClinicalTrials.gov specifying an English language requirement and only 2.7% specifically mentioning accommodation of translation to another language. Compared to posted protocols, the information in ClinicalTrials.gov under-reported both English language requirements and accommodations for translation. There are a number of things that could be done to further strengthen the paper, both in terms of the results presented and the discussion.

*Response*:

We appreciate Reviewer 2’s enthusiasm for the research. Thank you.

2. It would be particularly helpful, and a greater contribution to the literature, if the authors could demonstrate a relationship between accommodation for translation and actual increased diversity in clinical trials. While this would not be possible for all languages, it seems like it should be possible to search information on ClinicalTrials.gov to determine whether accommodation of translation resulted in overall greater proportions of non-white participants. Another approach would be to examine the relation between accommodation of Spanish translation and the proportion of Hispanic participants. This information would provide the missing link in the mechanism linking translation to participation.

*Response*:

We did not undertake the analysis as suggested by the reviewer (“it seems like it should be possible to search information on ClinicalTrials.gov to determine whether accommodation of translation resulted in overall greater proportions of non-white participants.”) As the reviewer appreciates, the majority of non-White participants by race (e.g., Black/African-American, American Indian or Alaska Native, Asian, and Native Hawaiian or Other Pacific Islander) do speak, read, or understand English. Further, ethnicity data are an inadequate proxy variable for language proficiency; many individuals who are Hispanic or Latino are proficient in English, and many individuals who do not identify as Hispanic or Latino are not proficient in English. One might argue that establishing a meaningful connection between language requirements and the proportion of Hispanic or Latino participants enrolled in a clinical trial would also require understanding the proportion of Hispanic or Latino participants eligible for enrollment at a given site. We do not have enrollment by site available on Clinicaltrials.gov to analyze this correlation.

That said, we agree completely with Reviewer 2’s assessment that the impact of this manuscript would be increased by demonstrating a relationship between accommodation for translation and increased diversity in clinical trials. Per Reviewer 2’s suggestion, we executed an analysis of the relationship between accommodation of Spanish translation and the proportion of Hispanic or Latino participants in a clinical trial. Of the 14,367 clinical trials that we reviewed in 2019 and 2020, only 323 had posted results on ClinicalTrials.gov. Of the 323 trials that had posted results, only 189 reported ethnicity data, and of those 189 trials, only 4 were identified as accommodating Spanish. The small sample makes it impossible to draw any meaningful conclusions of the relationship between accommodation for translation and increased diversity in clinical trials. We do plan to undertake a long-term study to address this issue.

We have edited the manuscript to respond to Reviewer 2’s suggestion. We note how the small sample size impeded this analysis.

Excerpt:

Methods: added:

In order to establish a relationship between the accommodation of a non-English language and increased diverse representation in clinical trials, we reviewed the ethnicity data in the trials that had posted results to ClinicalTrials.gov.

Non-English Language Accommodation and Clinical Trial Enrollment

To determine whether accommodation of non-English language correlated with an increase in the ethnicities of research participants, we reviewed trials with posted results. Of the 14,367 clinical trials initiated in 2019 and 2020, only 323 had posted results on ClinicalTrials.gov. Of the 323 trials that had posted results, only 189 reported ethnicity data, and of those 189 trials, only 4 were identified as specifically mentioning accommodation of a non-English language. The small sample size of completed studies with posted results impeded any correlative analyses on non-English language accommodation and the ethnicities of research participants.

3. Among the possible accommodations for translated documentation, it seems like a missed opportunity not to comment on the potential for using the consent short form (21 CFR 50.27).

*Response*:

Reviewer 2 correctly identifies an opportunity to comment on the use of short forms as an accommodation. We have added this discussion point to the final paragraph of the manuscript that discusses measures for accommodating individuals who do not speak, write, or comprehend English. In addition to contributing to the depth of this section, the point on short forms emphasizes the relevance of this manuscript— ~19.0% of clinical trials in 2019 and 2020 are failing to even use short forms to include individuals who are proficient in non-English languages. We do not, however, want to end with the recommendation that the use of the short form is sufficient. The Common Rule and FDA regulations both require *“*information that is given to the subject or the representative shall be in language understandable to the subject or the representative,*”*^[1]^ and the short form cannot contain sufficient information for the individual to understand the research study. The FDA advises, “When the study subject population includes non-English speaking people or the clinical investigator or the IRB anticipates that the consent interviews will be conducted in a language other than English, the IRB should require a translated consent document to be prepared and assure that the translation is accurate.”^1^ There are of course practical resource concerns that limit translation of documents.

Excerpt: (Discussion)

Use of a short form consent document summarizing the basic elements of informed consent in a non-English language does not offer study specific information. It may be used as a method to document that the elements of informed consent were presented orally, but optimally should be a temporary fix. Future work should delineate the conditions under which use of a short form consent document is appropriate and the conditions under which translation of the informed consent and other study materials is necessary or expected.

4. An additional important point for inclusion of non-English speaking participants in trials is to modify the incentives and disincentives for sites to include these participants. These include covering not only the translation costs, but the costs associated with use of interpreter services and of added staff time required for visits conducted using an interpreter.

*Response*:

We agree with Reviewer 2’s suggestions and have mentioned these suggestions in the discussion of the manuscript. Of note, we set out to write a data-driven paper that establishes a baseline understanding of the frequency of English requirements in clinical trials, and we do not intend to provide a comprehensive list of policy recommendations in this manuscript. Additional work is needed to construct these recommendations, and we have already initiated a survey of Institutional Review Board policies regarding the accommodation of non-English languages in clinical trials in an effort to understand the current landscape of policies for accommodation.

Excerpt (Discussion):

Institutions and investigators with access to translation services for clinical purposes could extend the services to research, and provision of interpreter and translation resources might incentivize inclusion of diverse populations.

5. There should be at least mention of the distinction between clinical trial sites potentially being able to offer staff fluent in some languages (e.g. Spanish) and clinical trial sites with truly broader language capacity (e.g. through interpreter services), which could accommodate a much wider range of languages. Stated another way, the way this analysis is presented, all trials would be 'inclusive' if they just included Spanish - which may be easier to achieve. However, true equity in access to trials will require moving beyond a single additional language.

*Response*:

We appreciate Reviewer 2 directing our attention to this. Although we do not refer to the 2.7% of trials that accommodate another language as ‘inclusive,’ it is tempting for the reader to assume that they are inclusive since they are discussed in conjunction with the 18.98% of trials with English requirements that we designate as ‘exclusive.’ We have highlighted this nuance and echoed Reviewer 2’s call for equity in the Discussion.

Excerpt:

As a related point, it should not be assumed that the clinical trials categorized as ‘accommodates translation’ are sufficiently inclusive. Any trial that accommodates at least one other language was categorized as ‘accommodates translation,’ but just and appropriate inclusion often requires accommodation of more than a single additional language.

REVIEWER 3

1. This is a timely and important topic, and I commend the authors for submitting it to a systematic analysis. It is to be hoped that studies such as this spark a trend that will bring more equitable representation in clinical research. Below are specific comments

Response:

We appreciate Reviewer 3’s enthusiasm and share their hope to spur more equitable representation in clinical research.

2. Abstract: the percentages under the results section do not add up to 100, clarify that the rest of the percentage were not eligible for inclusion, did not specify language, etc

Response:

We have incorporated this suggestion will clarify the results presented in the Abstract.

Excerpt:

The remaining trials in this analysis and the following sub-analyses did not mention English requirements or accommodation of languages other than English.

3. Are the phrases "ability to read, speak, and/or understand English", "English language proficiency", "required English", "required English language", and "mandate English" interchangeable? Similarly, "who cannot read, speak, and/or understand English" and "are non-Native English speakers" can mean widely different things. Consistent terms should be used in the abstract. A definition of Limited English Proficiency (LEP) is offered in the introduction Perhaps the larger issue is that protocols do not have a consistent language to classify proficiency and that is also something that needs to change (this point is made on page 8, but the abstract uses the terms interchangeably).

*Response*:

Reviewer 3’s comment is well-taken. We have changed the language in the Abstract to be consistent, referring broadly to English language requirements and requirements for English proficiency and not conflating requirements to read, speak, and/or understand English.

We also changed the word ‘mandate’ to the word ‘require’ throughout the Abstract and the manuscript, since these words have slightly different meanings, and ‘require’ more accurately captures what we mean when discussing English language requirements.

Abstract Excerpts:

We sought to establish the frequency with which English language is required

Systematic exclusion of individuals with LEP from clinical trials

Manuscript Excerpts:

We sought to establish the frequency with which English is required

It is surprising that federally funded trials were not only more likely to require English

4. Page 2, Line 37-38 belongs in the conclusion (not introduction)

*Response*:

We agree and have deleted this sentence from the introduction. We did not re-paste it in the conclusion since we believe the conclusion already conveys this idea.

Excerpt:

~~The requirement for English proficiency across interventional trials limits appropriate representation, diversity, and inclusion in clinical trials.~~

5. Page 2, Line 41-42 - what does "varying time points" mean?

*Response*:

We use the phrase ‘varying time points’ to refer to the fact that some of our searches in ClinicalTrials.gov were restricted to 2019 and 2020 and some identified trials prior to 2019. We have edited the Methods section to specify the time range for each search performed in ClinicalTrials.gov. We also deleted the phrase ‘varying time points’ from the Methods and Findings section of the Abstract.

Excerpt:

Lines 41-46: We used the advanced search function on ClinicalTrials.gov to search for interventional studies for adults (18-64) and older adults (65+) with at least one site in the United States with an actual start date between 01 January 2019 and 01 December 2020. We performed additional searches for interventional studies for adults (18-64) and older adults (65+) with at least one site in the United States across four therapeutic areas (depression, diabetes, breast cancer, and prostate cancer).

Abstract Excerpt:

We used the advanced search function on ClinicalTrials.gov specifying interventional studies for adults with at least one site in the United States ~~across varying time points~~.

6. Page 6, Line 111: The term Latinx has gained popularity in academic circles, but it is less known, widespread and accepted in non-academic communities in the United States that might be expected to identify as "Latinx" (very few people actually do). It is especially important to select terms carefully or perhaps acknowledge the complexities of using rarified academic language in an article concerned with increasing representation.

*Response*:

We have replaced ‘Latinx’ with ‘Hispanic and Latino.’

Excerpts:

…infection in the Hispanic and Latino populations in the United States

…and severity in Hispanic and Latino populations

7. Page 11, Lines 201-208: The last paragraph outlines some general suggestions, but it would be a stronger conclusion if the authors specifically provided a set of concrete recommendations to increase language (and other kinds) of representativeness. A good place to start to look into such guidelines might be literature on community-based research and patient-centered clinical research (PCORI, a federally-funded organization does just this).

*Response*:

We completely agree with Reviewer 3 that the next step in this work will be identifying concrete recommendations to increase language and other kinds of representativeness in clinical trials. As mentioned in our response to comment 4 by Reviewer 2, however, we believe that concrete recommendations should be generated through a formal analysis, and we have taken steps to initiate this analysis. We do not intend to offer more concrete recommendations until this formal analysis is complete.

8. Trials that do not require English may already be exclusive "by default" - the next step would be looking at actual recruitment for those trials vs. the ones that are English-exclusive. I would imagine that the differences in the proportion of LEP subjects would be negligible and that the only significant differences would be in those trials that actively recruit non-English speakers.

*Response*:

Reviewer 3 makes an excellent point, but we do not presently have the ability to execute this analysis. First, data on the language spoken by research participants are not readily available. Second, determining whether a clinical trial is exclusive to non-English languages (“by default”) requires knowledge of the languages spoken in the population eligible for a clinical trial at a given site. A trial that fails to enroll individuals who are not proficient in English, for example, cannot be determined to be exclusive if the only eligible population at the trial site are proficient in English.

9. It is also important to acknowledge that English proficiency requirements are only part of the story, it is equally important for clinical research to actively seek recruitment from populations that are underrepresented due to other socio-economic factors. The COVID-19 crisis underscores the importance of inclusivity in clinical research: in the US, rates of vaccination are much lower in black and Hispanic populations than in whites. In order to understand why this is the case and more urgently, begin to close those gaps, inclusive research is essential.

Response:

We could not agree more with Reviewer 3’s comment. We recently led a multi-year project on diversity, inclusion, and equity in clinical research, culminating in the August 2020 publication of a comprehensive [guidance document](https://mrctcenter.org/diversity-in-clinical-research/guidance/guidance-document/) and [toolkit](https://mrctcenter.org/diversity-in-clinical-research/tools/toolkit/) on the topic. In March 2021, we published an article entitled, “Justice, diversity, and research ethics review,” in Science.^[2]^ While we appreciate that language is only one dimension of diversity that merits consideration when striving for inclusive and representative research, we intend to keep the scope of this manuscript focused on language requirements.

10. Finally, it may be somewhat out of the scope of this paper, but the lack of translation resources for LEP researchers in other settings (particularly low and middle-income countries) also means that research not conducted in English has few opportunities to be funded or published by major organizations. It may be worthwhile to mention that journals and funding agencies could do more to facilitate research with a global impact, which is particularly important given the global epidemic.

Response:

While outside the scope of this paper, we completely agree with this comment.

11. It is perhaps somewhat understandable that researchers simply do not have the time, training or capacity to conduct outreach efforts that include medically-underserved populations. However, without those efforts, clinical research will increase existing inequalities. Until clinical research makes a real effort to include underrepresented populations, we cannot expect medicine and healthcare to have equitable impact.

Response:

Again, we agree with Reviewer 3’s assessment of the issue.

We hope and believe that the revisions have strengthened the manuscript, and we thank you for the comprehensive reviews. We are happy of course to work with you further to meet the expectations of *PLOS Medicine,* and we look forward to hearing from you.

Sincerely,

Barbara Bierer, MD, on behalf of the authors.

[signatures]

1. <https://www.fda.gov/regulatory-information/search-fda-guidance-documents/guide-informed-consent#content>
2. Strauss DH, White SA, Bierer BE. Justice, diversity, and research ethics review. Science. 2021 Mar 19;371(6535):1209-11. <https://science.sciencemag.org/content/371/6535/1209.summary>.
