# Supplementary material for: Evaluating the frequency of English language requirements in clinical trial eligibility criteria: A systematic analysis using ClinicalTrials.gov
Source: PLoS Med. 2021 Sep 14;18(9):e1003758. doi: 10.1371/journal.pmed.1003758 (PMC8439488; doi:10.1371/journal.pmed.1003758)
Supplement: Attachment — Submitted filename: 2021-07-23 PLOSMed response letter.docx [file pmed.1003758.s003.docx]

Response to *PLOS Medicine*

Manuscript ID PMEDICINE-D-21-01563R2

To the Editors and Reviewers,

Thank for your further careful consideration of our manuscript, and for your enthusiasm for publishing the paper providing certain editorial and publication questions are addressed. In the accompanying manuscript, we have added p values and citation dates, as requested, and we have uploaded a version of the edited manuscript both in track changes and as a clean copy. There was only one substantive comment which we address below.

One quick comment: you requested the revised manuscript in one week, but then gave us a resubmission date ~24 hours after we received the actual email. We presume that the date July 16 11:59 PM was in error, as it took us the weekend to address the comments.

Direct quotes from the editors and reviewers are numbered followed by the authors’ *Response*.

EDITORS

1) Please include p-values for all estimates provided in the results section - text and tables. For instance, tables 2 & 3 have 95% CIs but no p-values

*Response:*

We have had an email exchange offline about this request from the editors. Today we received the following from Beryne Odeny, Editor, on behalf of the journal:

Thank you for your email re: p-values. Your reasoning and explanation for opting not to include p-values make sense. Many thanks for the clarification.

Please ignore the request for p-values and upload your manuscript at your earliest convenience

We have not, therefore, added the p-values.

2)	References – please consistently include access dates for weblinks e.g., refs #1, #6, #9

*Response:*

We have updated the references appropriately.

.

REVIEWER 2

The revised paper provides thoughtful responses to the reviewer critiques, and has made edits and additions that strengthen the manuscript.  My one additional comment on this read is that it would be helpful to know more about the characteristics of the 390 trials that specifically mentioned accommodation of translation to another language.  If the reasons for affirmative accommodation of translation are related to geography, specific conditions likely to occur in non-English-speaking populations, or other identifiable correlates, this would be an important additional finding that would help readers to better understand how language requirements in clinical trials are being handled.

*Response*:	Table 1

Reviewer 2 suggests an additional analysis. We have downloaded the data as available from ClinicalTrials.gov. In this small sample, we cannot readily find meaningful characteristics across phase of trial, therapeutic area, intervention, or other that we wish to put forward to the general readership. There are several reasons for our hesitancy, including the lack of a reliable denominator for comparison. For instance, we examined phase of trial: Of 390 trials, 301 listed Phase of trial as “not applicable.” Of the 89 trials that listed a phase of trial, there was no clear predominance (see Table 1), and any inference of preferential accommodation of language translation by phase would need to account for the number of trials by phase submitted in that period of time, by site, by therapeutic area (since we know through our submitted data that language requirement differs by therapeutic area.) When we analyzed therapeutic area (Table 2), no pattern emerged, particularly since we do not have an appreciation of the denominator. Similarly, geography is complex: while the lead institution is readily available, additional collaborating institutions are not. That data would require a manual analysis (finding each ClinicalTrials.gov entry, manually listing all collaborating sites, then identifying their principal geography), and that manual analysis would not be informative without zip code and demographic data for the catchment area of the site. Even the lead institution may not reflect the institutions that are recruiting. Having spent significant time with this data and with the information available on Clinicaltrials.gov, we cannot speculate as requested by Reviewer #2, although we agree that this would be an interesting empirical question (subject to appropriate data availability).

Three other issues to address:

1. We are happy to have an uncorrected proof published online ahead of print. We would prefer for this current version of the manuscript be published rather than earlier versions of the manuscript. We think that the revisions suggested by the reviewers and editors have significantly strengthened and clarified the manuscript.
2. We believe that the reviews themselves need not be published, as we think they will not enhance clarity compared to reading the final version of the manuscript.
3. Author contributions.

I could not find a form for each author to “fill in” as stated in your email.

As lead and corresponding author, I have requested the two co-authors to identify their contributions, and I have added my own according to the CRediT Taxonomy.

**Akila Muthukumar**

Roles: Conceptualization, Data curation, Formal analysis, Methodology, Software, Writing—original draft preparation, Writing—review and editing.

**Walker Morrell**

Roles: Conceptualization, Data curation, Formal analysis, Methodology, Project administration, Writing—original draft preparation, Writing—review and editing.

**Barbara Bierer**

Roles: Conceptualization, data curation, formal analysis, methodology, project administration, supervision, visualization, writing-review and editing.

We hope and believe that this further clarification is helpful, and we thank you for the comprehensive reviews. We are happy of course to work with you further to meet the expectations of *PLOS Medicine,* and we look forward to hearing from you.

Sincerely,

Barbara Bierer, MD, on behalf of the authors.
